# Environmental Conditions during Breeding Modify the Strength of Mass-Dependent Carry-Over Effects in a Migratory Bird

**DOI:** 10.1371/journal.pone.0077783

**Published:** 2013-10-15

**Authors:** Xavier A. Harrison, David J. Hodgson, Richard Inger, Kendrew Colhoun, Gudmundur A. Gudmundsson, Graham McElwaine, Tom Tregenza, Stuart Bearhop

**Affiliations:** 1 Centre for Ecology and Conservation, University of Exeter, Penryn Campus, Cornwall, United Kingdom; 2 Institute of Zoology, Zoological Society of London, London, United Kingdom; 3 Wildfowl and Wetlands Trust, Comber, County Down, Northern Ireland; 4 Icelandic Institute of Natural History, Reykjavik, Iceland; 5 Downpatrick, County Down, Northern Ireland; University of Missouri-Columbia, United States of America

## Abstract

In many animals, processes occurring in one season carry over to influence reproductive success and survival in future seasons. The strength of such carry-over effects is unlikely to be uniform across years, yet our understanding of the processes that are capable of modifying their strength remains limited. Here we show that female light-bellied Brent geese with higher body mass prior to spring migration successfully reared more offspring during breeding, but only in years where environmental conditions during breeding were favourable. In years of bad weather during breeding, all birds suffered reduced reproductive output irrespective of pre-migration mass. Our results suggest that the magnitude of reproductive benefits gained by maximising body stores to fuel breeding fluctuates markedly among years in concert with conditions during the breeding season, as does the degree to which carry-over effects are capable of driving variance in reproductive success among individuals. Therefore while carry-over effects have considerable power to drive fitness asymmetries among individuals, our ability to interpret these effects in terms of their implications for population dynamics is dependent on knowledge of fitness determinants occurring in subsequent seasons.

## Introduction

A central challenge in population ecology is identifying the factors that drive variation in observed reproductive success among individuals. Reproductive success can be expressed as a function of both processes occurring within the current season, such as summer climate affecting hatching success [1], as well as processes from previous seasons whose effects have persisted into the current time period, so-called ‘carry over effects’ [2,3]. Carry-over effects have been shown to be powerful drivers of fitness asymmetries among individuals [4], and have been described in numerous taxa including birds, mammals and reptiles (reviewed in 3). For example, carry-over effects can be mediated by body mass prior to reproduction, where individuals that have experienced superior resource access in the season before breeding have higher body mass during the breeding season, and consequently are able to invest more in reproduction [5,6]. Mass-dependent carry-over effects are likely to be particularly pronounced in migratory capital-breeders [3,7], which must accrue resources prior to the breeding season to fuel both travel to the breeding grounds and reproduction upon arrival. Accordingly there is a wealth of evidence demonstrating mass-dependent carry-over effects in Arctic-nesting bird species, where differences among individuals in rates of pre-migration mass storage have been linked to variation in both migratory timing [8] and probability of breeding [5,6,9,10].

While evidence for the presence of carry-over effects in a multitude of taxa is growing, only recently have studies begun to investigate how the strength of carry-over effects from prior to breeding may interact with, and be modified by, processes during the breeding season to influence reproductive success [4,10]. It is difficult to quantify the potential for carry-over effects to drive variance among individuals in their reproductive success without also quantifying the potential for the strength of those effects to be magnified or reduced by events occurring during breeding. Legagneux et al. [10] provided evidence of mass-dependent carry-over effects in Greater Snow Geese (*Anser caerulescens atlanticus*), but also demonstrated that the advantage of increased body mass for reproduction could be negated in some years by favourable environmental conditions during breeding. Though we may infer that the strength of interaction between pre-breeding body mass and environmental conditions during breeding is likely to vary among years [10], the direction(s) in which they act may not be universal among species. Empirical evidence describing the strength and form of these interactions between separate periods of the annual cycle is lacking, and the manner in which they affect demographic processes remains poorly understood, largely because such estimates require that we track individuals between seasons within and among years [4,11-13]. While the influence of environmental conditions during breeding on reproductive success in Arctic-nesting species is fairly well characterised [1,14,15], past studies have measured reproductive success at the population level by counting proportion of juveniles in flocks the season following breeding. Population-level measures preclude the quantification of carry-over effects, which are by definition an individual-level phenomenon [2,3]. Without individual level data (e.g. pre-breeding mass, migratory departure date, or number of offspring produced by a single individual), one cannot readily discern whether the observed the *per capita* breeding output of the population is the product of either carry-over effects, or density-dependent seasonal compensation [2,16], both of which have different implications for our understanding of the mechanisms that regulate population dynamics. 

When investigating the interaction between carry-over effects from prior to breeding and environmental conditions during breeding, there are two broad hypotheses whereby such interactions may occur: i) in years of poor environmental conditions during breeding, only those individuals with largest amount of endogenous energy reserves are likely to breed [10]; or ii) only in years of favourable breeding conditions are the asymmetries in reproductive success among individuals of high and low body mass fully realized, because only these years provide an opportunity for individuals to utilize stored mass to finance reproduction. Conversely, it may be the case that there is no interaction between two processes occurring in season *t* and season *t*+1 respectively, and as such they simply operate in an additive and independent manner. Here we combine 6 years of data on body mass in the season prior to breeding and environmental conditions during breeding to examine how mass-dependent carry-over effects and environmental conditions may interact to influence reproductive success in an Arctic-nesting migratory bird, the light-bellied Brent goose (*Branta bernicla hrota*). 

## Methods

### Ethics Statement

In the UK all work was carried out under UK home office licence, Environment and Heritage Service (NI) wildlife licence and BTO cannon netting permits. In Ireland all work was carried out under National Parks and Wildlife Licence and BTO cannon netting permits. In Iceland work was carried out in conjunction with the Icelandic Natural History Society regulations. All field procedures were approved by the University of Exeter Ethics and Health and Safety Committees. All work was carried out with land owners' permission.

### Study Species and Data Collection

The East Canadian High Arctic (ECHA) population of light-bellied Brent geese (*Branta bernicla hrota*) overwinters around the coast of Ireland from late-August to April, subsequently staging for one month on the west cost of Iceland before breeding in the Canadian Arctic [17]. Brent geese feed preferentially on high-quality marine resources such as *Zostera* spp., and green algae (*Enteromorpha* spp. and *Ulva lactuca* L), but also shift to lower quality terrestrial grassland during the overwinter period once the density of the preferred food sources has diminished beyond a level that is no longer profitable to exploit [6].. Satellite telemetry data has shown that mean arrival date in the Canadian Arctic breeding grounds is 1^st^ June (K. Colhoun & G. Gudmundsson, unpublished data), with clutch initiation occurring roughly 7-10 days after arrival [18]. Modal clutch size is 4 eggs (range 2-6; [18]).

The Irish Brent Goose Research Group and collaborators have marked over 3500 light-bellied Brent geese to date from across the entire range (Ireland, Iceland and Canada). In both Ireland and Iceland, geese were caught in cannon nets while in Canada flightless adults (during moult) and juveniles were herded into enclosures. Birds were sexed either by cloacal examination, or using molecular markers as described in Harrison et al. [19], fitted with individually-coded colour leg rings and had morphometric data collected (mass, wing length and skull length). The resighting database currently contains over 95,000 records of marked birds, many of which include information on adult associations (breeding pairs) and number of juveniles in a family group. Previous work has verified that these familial associations represent parents and true genetic offspring [20]. Details of assignment of family groups and breeding pairs can be found in Inger et al. [6]. Our analyses used 6 years of data from 213 female light-bellied Brent geese for which we could quantify breeding success in the year of capture when mass was measured by counting the number of offspring they returned to wintering grounds with the following year. Of these, 92 females were observed to return with offspring the year after capture. The remaining 121 birds did not successfully breed in the year of capture, returning to the wintering grounds without offspring. Birds were only assigned as non-breeders if there were 3 or more records for the year after capture where they had been recorded without juveniles, and if they had been recorded in the year of capture as having an adult associate, to avoid noise caused by assigning potential singletons as non-breeders.

### Body Mass Data

We calculated the Scaled Mass Index (SMI,[21]) from our morphometric data of body mass and skull length of 468 adult females captured during Icelandic spring staging (April-May). The SMI scales the mass of all individuals to that expected if they were all of identical body size. We scaled all birds to the mean skull length (89.6mm), using a Secondary Major Axis (SMA) slope of 3.4, calculated as the ratio of the slope of a least squared regression of log(Mass) on log(Body Size) (1.003) to the correlation coefficient between those two variables (0.29, [21]). We note that this is not a measure of body condition based on mass-length residuals, which have been heavily criticised [22], but a metric that standardises mass among individuals based on an inherent power law between mass and size calculated from the data [21]. As with many capital breeders, light-bellied Brent geese show strong non-linear seasonal trajectories in body mass, whereby they rapidly increase size of fat stores prior to migration to fuel migratory flight and and finance reproduction [6]. We therefore corrected the point estimates of scaled body mass index for seasonal trajectory by fitting a 2^nd^ order polynomial term for day of annual cycle ( F_2,465_ = 122.5, p<0.001, r^2^ = 0.34). Residuals of these models were taken and added to the mean mass for females in the sample (1464.2g) to provide a seasonally corrected mass estimate, independent of body size, for each female to be used in subsequent analyses [10], which we refer to hereafter as ‘mass’. We extracted the corrected mass estimate for the 213 females for which we could confidently assign reproductive status the year after capture (see above) to use in subsequent analyses. There was no evidence that mean mass differed by year in our sample (Figure S1 in File S1). 

### Environmental Data

We obtained data for the North Atlantic Oscillation (NAO) Index from the Climate Prediction Centre [23], and extracted the monthly mean values for June. We used NAO for June as this is the period when Brent geese nest and lay clutches [18]. The June NAO data showed a significant non-linear temporal pattern (2^nd^ order polynomial for Year, F_2,60_= 5.82, p=0.005; Figure S2 in File S1), and so we de-trended the data by taking the residuals of the non-linear model of June NAO over time. If there is a true causal relationship between variables, then the residuals should still be correlated independently of any correlation between the original variables [24]. We used the de-trended residuals for June for each breeding cycle from 2004/5 to 2009/10 in our analysis.

The NAO index is representative of weather conditions throughout the Brent goose’s breeding range, and its ecological effects are well characterized [25,26]. Large-scale climatic predictors such as the NAO have often been shown to have superior explanatory power compared to local environmental variables such as rainfall or temperature [27], and indeed have been shown to correlate with local weather conditions in the Canadian Arctic [15]. An additional advantage of the NAO index is that it is a composite proxy measure of multiple non-independent climatic variables including rainfall, temperature and winds, and so its use in predictive models avoids the potential problems of autocorrelation among multiple explanatory variables [26]. Positive NAO indices are generally associated with intense low pressure over Iceland [26] and as a result an increase in the severity of westerly winds, storms and precipitation in the Arctic [25,26]. Conversely, negative NAO values generally represent favourable environmental conditions. Values from June represent conditions during breeding, as it is at this time that light-bellied Brent geese initiate nesting and lay clutches [18]. Several recent studies have found that large scale climatic predictors such as the North Atlantic Oscillation (NAO), when used as a proxy for local environmental conditions, have a significant effect on the reproductive success of Arctic-nesting bird species [14,15]. Mechanisms by which environmental conditions are expected to influence reproductive success include: i) lower temperatures causing reduced juvenile survival/growth due to increased thermoregulatory costs and/or reduced food availability [1,15],, ii) poor pre-breeding conditions affecting food availability/ability to sequester endogenous capital for breeding [28]; iii) poor weather increasing migration costs, which can also reduce the capital available for breeding [29]; and iv) longer migration times due to delays caused by poor weather reducing the time available to successfully raise young before the return of unfavourable late-summer environmental conditions, or causing a mismatch between peak offspring nutritional requirements and food availability [30].

### Statistical Analysis

All models were fitted in the software R v2.15 [31]. We used a GLMM with Poisson errors and log link to investigate variables affecting number of offspring produced, using the package ‘lme4’ [32]. We evaluated the support in the data for 8 competing models designed to explain variation in reproductive success as a function of carry-over effects from staging body mass, summer environmental conditions, or a combination of both. (Table 1). To account for unequal sample sizes among years we included year of measurement as a random intercept term in the models. All variables were z-transformed prior to analysis to have mean 0 and standard deviation of 1 to put all predictors on a common scale and make main effects interpretable in the presence of interactions [33]. Model selection was performed using an information-theoretic approach, using the R package ‘MuMIn’ [34] to rank all models based on AIC_C_. We considered all models within Δ6 AICc units of the top model as the best supported models, but also applied the ‘nesting rule’ [35,36] whereby models in the Δ6 AICc set were not retained if they were more complex versions of nested (simpler) models with better AICc support (higher up in the table). The nesting rule prevents the retention of overly-complex models that do little to improve the fit to the data [37]. Those models present in the Δ6 AICc set after application of the nesting rule were selected for model averaging using the function ‘model.avg’ in the MuMIn package. 

**Table 1:** Eight competing models investigating the factors affecting the reproductive success of light-bellied Brent geese, measured as number of offspring females returned to the wintering grounds following Autumn migration from the breeding quarters in the Canadian High Arctic.

**Model**	**Predictors**	**Prediction**
1	Mass	Winter body mass (carry-over effect) affects number of offspring produced, irrespective of environmental conditions during breeding
2	NAO	Only environmental conditions during breeding affect reproductive success
3	Mass + NAO	Mass affects reproductive success (carry-over effect), but the intercept of the relationship changes based on environmental conditions during breeding
4	Mass * NAO	The slope of the relationship between mass (carry-over effect) and reproductive success changes depending on environmental conditions during breeding
5	Mass^2^	Mass affects reproductive success in a non-linear fashion (carry-over effect), and does not depend on environmental conditions during breeding
6	Mass^2^ + NAO	Mass affects reproductive success in a non-linear fashion (carry-over effect), but the intercept of the relationship between mass and number of offspring changes depending on environmental conditions during breeding
7	Mass^2^ * NAO	Mass affects reproductive success in a non-linear fashion (carry-over effect), but the non-linear slope changes depending on environmental conditions during breeding
8	Null	Intercept Only Model

‘NAO’ is the North Atlantic Oscillation index for June, and is representative of environmental conditions on the breeding grounds in early summer, where nesting is initiated and clutches are laid.

We present model-averaged predictions from these models alongside predictions from the top model parameterised under a Bayesian framework, which gives predicted means and 95% credible intervals that are exact for the given sample size [38]. R code for these models is available in the Supporting Information (Code S1 in File S1). We calculated r^2^ for the top model using the methods detailed in Nakagawa & Schielzeth [39] for calculating r^2^ for mixed models. 

#### Multivariate Models

The use of derived variable analyses (i.e. correcting body mass for seasonal trends) using best linear unbiased predictors (BLUPs) has been criticised for its anti-conservatism [40]. To test the robustness of our results, we verified our analyses using a multivariate mixed model framework. The advantage of such an approach is that it allows the estimation of the posterior correlation between mass and reproductive success (the carry-over effect), while simultaneously controlling for confounding effects such as body size, or when individuals were measured, and evaluating support for predictors such as June NAO (summer environmental effects). It thus prevents the need to use predicted values from prior models in subsequent models, which can cause bias in results [40]. We used the R package ‘MCMCglmm’ [41] to fit a bivariate response model with number of juveniles and mass (raw mass at capture) as Poisson- and Gaussian-distributed responses, respectively. We modelled mass as a function of both time (2^nd^ order polynomial, as above in ‘Body Mass’ section) and skull size. We modelled number of juveniles as a function of June NAO. We then estimated the posterior correlation between mass and juveniles following Harrison et al. [42], whereby a significant positive correlation (95% credible intervals do not cross zero) is representative of a carry-over effect from winter mass after controlling for other confounding factors. Models were run for 250000 iterations after a burn in of 50000 and used a thinning interval of 50. The posterior correlation between Mass and Number of Juveniles was calculated from the stored values in the Markov chain as the posterior mode of the covariance (Mass,Number of Juvenilies) divided by the product of the standard deviations of Mass and Number of Juveniles. All R code for these models and posterior calculations is available in Supporting Information (Code S2 in File S1). 

## Results

### Factors Influencing the Number of Offspring Produced

**Figure 1:**
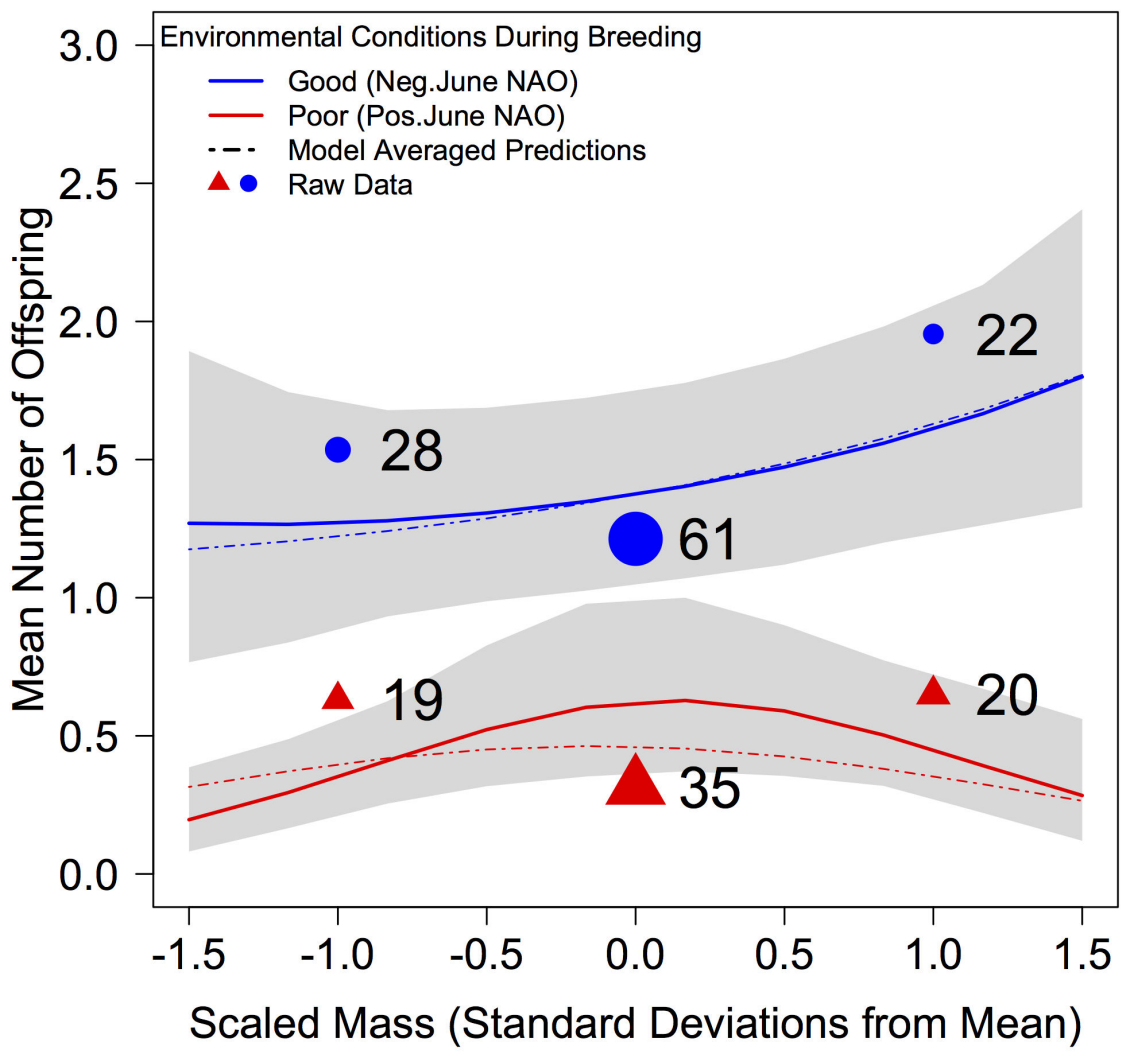
Graph illustrating how the strength of mass-dependent carry-over effects is modulated by the environmental conditions during breeding. In years with positive June NAO (red line), representative of poor weather conditions during breeding, higher body mass does not yield an increase in reproductive success. Conversely, when breeding conditions are favourable (Negative June NAO, blue line), individuals with greater body mass return the following year with more offspring. The x axis represents body mass (as Scaled Mass Index) expressed in units of standard deviations from the mean, where ‘0’ represents an individual of average mass in the sample. Shaded areas span the 95% credible intervals for the fitted means. Points are raw data means for years with positive June NAO (triangles) and negative June NAO (circles), averaged over 1 SD bins of body mass and plotted at the midpoint of that bin. Point size is proportional to sample size per bin. Sample size per bin is displayed next to each point. Predictions for both good and poor environmental conditions are for ‘average' conditions in each scenario, being the mean of years with negative NAO residuals for ‘good’ years and mean positive residuals for ‘bad’ years. Note that as the data predict over values within 1.5 SD of mean body mass, some data at the tails of the distribution for body mass are excluded from the plot.

There were 4 models in the Δ6 AICc candidate set (Table 2). The best-supported model contained a terms for a quadratic effect of body mass prior to migration to breeding, June NAO (representative of environmental conditions during breeding) and their interaction. In years of favourable environmental conditions during breeding (negative June NAO), individuals of higher body mass have much greater reproductive success than lower mass birds. However in years of poor breeding conditions, the advantage of higher body mass is largely negated (Figure 1), suggesting the strength of the carry-over effect is greatly reduced.

**Table 2:** Eight models investigating the factors that predict variation in reproductive success among light-bellied Brent geese, ranked by AICc.

**Int.**	**Mass**	**NAO**	Mass*NAO	**Mass^2^**	Mass^2^*NAO	**k**	**logLik**	**AICc**	**ΔAICc**	**weight**	**Retained**
-0.09	0.11	-0.58		-0.14	-0.27	6	-169.018	350.4	0	0.507	✓
-0.21	-0.01	-0.79	-0.29			5	-170.293	350.9	0.43	0.408	✓
-0.21		-0.79				3	-174.799	355.7	5.27	0.036	✓
-0.22	0.10	-0.81				4	-173.832	355.9	5.41	0.034	
-0.18	0.12	-0.80		-0.03		5	-173.625	357.5	7.1	0.015	
-0.45						2	-182.591	369.2	18.8	0	
-0.46	0.09					3	-181.778	369.7	19.23	0	
-0.41	0.12			-0.04		4	-181.503	371.2	20.75	0	

The best-supported model contained a quadratic effect of body mass prior to migration to breeding (Scaled Mass), June NAO (representative of environmental conditions during breeding) and their interaction. The interaction term has a negative coefficient, suggesting in years of favourable environmental conditions during breeding (Negative June NAO), the advantage of higher body mass is greatly increased. The dashed line separates the 4 models within 6 AICc units of the top model. We applied the ‘nesting rule’ [35] to these models, where models were not retained if they were more complex versions of nested (simpler) models higher up in the table (i.e. with better AICc support). Accordingly, only the top 3 models were retained. ‘Int.’: intercept, ‘k’: number of estimated parameters, ‘weight’: Akaike weight, ‘Retained’: indicates whether model was retained after applying the nesting rule.

Using the nesting rule, we retained only 3 models: the top model, a model containing a *linear* effect of mass and its interaction with June NAO (ΔAICc = 0.43), and a model containing only the effect of June NAO (ΔAICc = 5.27). Model averaged estimates from these three models are presented in Table 3. Predictions from the top model are presented in Figure 1 alongside model-averaged predictions. The marginal r^2^ (variance explained by the fixed effects) of the top model was 53%.

**Table 3:** Model averaged estimates for the 3 models in theΔ6 AICc top model set remaining after the nesting rule had been applied (see Table 2).

**Variable**	**MA Estimate**	**SE**	**Shrinkage**	**Importance**
Intercept	-0.15	0.11	-0.15	-
Mass	0.06	0.10	0.06	0.96
**NAO**	**-0.68**	**0.16**	-0.68	1
Mass*NAO	**-0.29**	**0.11**	-0.13	0.43
Mass^2^	-0.14	0.09	-0.07	0.53
Mass^2^*NAO	**-0.27**	**0.11**	-0.15	0.53

‘MA estimate’: Model averaged estimate, calculated using the ‘natural averages’ method [47]. ‘SE’: standard error for MA estimates. ‘Shrinkage’: model averaged estimates calculated using the ‘zeroes’ method where estimates are set to zero in models where they do not occur [47]. ‘Importance’: relative variable importance, calculated as the sum of the Akaike weights of the models in which that term appears in the top model set. Significant model-averaged terms are in bold.

### Multivariate Models

Results from the multivariate models were in agreement with the information theoretic approach (Table 4). Both a 2^nd^ order polynomial term for day of year and a linear term for Skull size significantly affected mass, and number of offspring was significantly influenced by June NAO. We observed a significant, positive posterior correlation between Mass and Number of Offspring (0.22, 95% credible interval 0.015 - 0.47, Table 4), after controlling for the effects of skull size, day of year and June NAO. This correlation is representative of carry-over effect of body mass, where individuals that are heavier prior to migration to breed are predicted to return to the wintering grounds with more offspring the following year. 

**Table 4:** Results from a Bayesian multivariate response model investigating the posterior correlation between body mass and number of juveniles.

**R-structure: ~us(trait):units**			
	**Mean**	**Lower 95% CI**	**Upper 95% CI**
Mass Variance	9038.08	7284.14	10756.58
Mass/N. Juveniles Covariance	21.23	0.58	43.47
Mass/N. Juveniles Covariance	21.23	0.58	43.47
N. Juveniles Variance	0.84	0.37	1.34
**Predictors**			
	**Mean**	**Lower 95% CI**	**Upper 95% CI**
Mass Intercept	-133.22	-552.20	293.83
N. Juveniles Intercept	-0.63	-0.94	-0.34
Mass:poly(cycleday, 2)1	2519.67	2276.07	2793.60
Mass:poly(cycleday, 2)2	413.29	143.92	676.31
Mass:Skull	21.95	16.94	26.57
N. Juveniles:June NAO	-0.94	-1.21	-0.64
**Posterior Correlation**			
	**Mean**	**Lower 95% CI**	**Upper 95% CI**
Mass / N. Juveniles	0.22	0.015	0.47

Concordant with the scaled mass index analysis, body mass was significantly affected by both skull size and a 2^nd^ order polynomial term for day of season (correcting for temporal trends in mass storage). June NAO significantly affected number of offspring, which supports the conclusions of our best-supported AIC model(s). Once controlling for these predictors, there was a significant posterior correlation between Mass and Number of Offspring produced, representative of a carry-over effect of body mass from spring staging.

## Discussion

Our results provide evidence that the reproductive success of light-bellied Brent geese is a function of carry-over effects driven by winter body mass, but that the strength of these carry-over effects is modified by environmental conditions during breeding. Individuals with higher body mass prior to migration to the breeding grounds have higher reproductive success than lower-mass birds, but only when the environmental conditions during breeding are favourable. Conversely in years of poor conditions during breeding, all individuals suffer a greatly reduced reproductive success, with no advantage of higher body mass. There are two important consequences of such a pattern: i) the advantage of accruing large endogenous resource stores prior to breeding fluctuates markedly among years in concert with the severity of environmental conditions during breeding; and ii) individuals will fail to realise their maximum potential reproductive success unless the years in which they accrue the largest body stores prior to migration also coincide with the years where the opportunity to utilise those stores during breeding is highest. Variance in reproductive success will therefore be greatest among individuals that consistently arrive at the breeding grounds in the best condition, and those that either arrive in consistently poor condition across years, or whose peak body mass mismatches the timing of the most favourable summer weather. The relative success of consistently carrying large fat reserves depends on the survival cost of carrying those stores in years of unfavourable weather where flight costs may be high [43]. In years of poor weather it may be that birds of intermediate mass are most successful [10], possessing sufficient energy stores to complete the migration and breed, but without suffering the increased flight costs that would result from excessively large fat stores. Light-bellied Brent geese cross the Greenland ice cap during migration from Iceland to the breeding grounds [17]; this route is 1000km shorter compared to having to fly around the ice cap, but the increased altitude requires such high flight muscle power output that Brent geese likely fly near or beyond the limits for aerobically sustained muscle performance [17]. Therefore in years of particularly poor weather it may be extremely disadvantageous to carry unnecessary fat stores that will only increase the demand on flight muscles during this phase of migration and severely constrain flight performance. 

The patterns we present here are consistent with previous, population-level studies that have examined the determinants of reproductive success in Arctic-nesting species. Ebbinge [44] found that in years where mean body mass of female Dark-Bellied Brent geese (*Branta bernicla bernicla*) prior to migration to breed was higher, breeding success, measured as the proportion of juveniles in winter flocks the following year, was also higher. Our results build upon those of Ebbinge [44] by quantifying the individual-level consequences of prior body mass by linking mass to number of offspring the following year. Madsen et al. [28] found that in years of high snow cover at the start of breeding, Pink-footed Geese (*Anser brachyrhynchus*) were forced to delay egg laying and suffered lower breeding success, indicating that individuals had to wait for suitable nesting sites to be exposed as the snow recedes, and subsequently the time in which they have to successfully rear offspring within the short Arctic summer was greatly reduced. Early nesting and clutch initiation is crucial to successful reproduction in Arctic-nesting species [28,45], and those individuals that arrive early on the breeding grounds usually have higher reproductive success [46]. However, late snowmelt can delay nesting for all birds [28,45] to the extent that there is no advantage to early arrival, and the low number of offspring produced in years of positive NAO most likely reflects the stochastic manner in which birds manage to secure nesting sites after poor weather conditions. During late-nesting years, higher female body mass is unlikely to confer any advantage in reproductive success, as survival prospects of offspring decline rapidly with lay-date, and geese most likely modify their clutch size based on the expected survival value of offspring given lay date [8]. Conversely, in years of favourable weather, those individuals that arrive early and with the largest mass stores will be able to nest early, as nest sites will most likely not be limited [28]. Early-arriving females are then able to maximise investment in clutch size and have sufficient time to rear offspring prior to the autumn migration back to the wintering grounds, explaining why we observe that in years of negative June NAO, those females with greater fat stores during winter produced more offspring.

Using a multivariate response model, we validated the results of our models run using a derived variable of mass corrected for body size and seasonal trajectory. We detected a significant, positive posterior correlation between mass and number of offspring produced, once controlling for body size and seasonal trajectory within a singular modelling framework. This correlation represents a carry-over effect of mass sequestered on the Icelandic staging grounds prior to migration to breed, in addition to the significant effect of June NAO that was also recovered by modelling number of offspring as a function of environmental conditions during breeding. As birds were measured once within years, and not multiple times across years, we lack the ability to estimate how the strength of the posterior correlation between mass and number of offspring fluctuates in concert with the severity of the environmental conditions experienced during breeding. The large credible interval around the correlation most likely result from constraining the model to estimate a single variance-covariance matrix for the mass and offspring variables across all years, where in fact it’s likely that the magnitude of this correlation varies considerably among years in a similar fashion to the patterns demonstrated in Figure 1. By employing multiple observations of individuals from across years, future work will focus on quantifying the degree to which posterior correlation between mass and number of offspring changes in concert with fluctuations in the magnitude of the June NAO. 

Our measure of reproductive success was the number of juveniles females returned to the wintering ground with in the year after capture. Therefore females in our sample recorded as having no offspring could have failed to breed successfully either because they i) failed to lay eggs, ii) successfully laid a clutch and reared offspring, but those offspring were subsequently lost to predation or died due to poor food availability prior to autumn migration [25], or iiI) lost offspring during the lengthy migration back to the wintering grounds. Stochastic mortality will add noise to our data, as for example a female with large mass stores may have laid a large clutch but subsequently lost all offspring during autumn migration, variation that would not be captured by our measure of either mass or NAO. While clutch size may be a more accurate reflection of carry-over effects from mass-dependent reproductive investment, our results are clearly robust to the stochastic mortality events outlined above, as we still detect carry-over effects from winter body mass. More importantly, our results represent a more accurate measure of true reproductive success, and reflect true variance in reproductive success among individuals within and between years that results from the interaction between carry-over effects and breeding conditions. 

 Our results suggest that in a migratory capital breeder, the advantage of higher capital stores for reproduction is not uniform across years, but instead varies according to environmental conditions experienced during the breeding season. The interaction we have described here between a carry-over effect from winter and within-season effects of weather during breeding are likely more common than currently reflected in the literature (but see 10), and highlight the importance of gathering individual-level data, both between seasons and among years. Seasonal interactions as described here are powerful drivers of fitness asymmetries among individuals [4], and knowledge of how processes from different periods of the annual cycle interact to influence reproductive success will have important implications for our understanding of the forces regulating the dynamics of animal populations.

## Supporting Information

File S1 **Online Supporting Information.** **Figure S1.** Body mass variation by year for light-bellied Brent geese calculated using 6 years of data. **Figure S2.** Temporal variation in June NAO from a 60 year dataset. **Code S1.** R code for Bayesian Hierarchical models used for prediction. **Code S2** R code for multivariate response model used to validate the derived variable analyses.
(DOCX)
