# Supplementary material for: Environmental Conditions during Breeding Modify the Strength of Mass-Dependent Carry-Over Effects in a Migratory Bird
Source: PLoS One. 2013 Oct 15;8(10):e77783. doi: 10.1371/journal.pone.0077783 (PMC3797109; doi:10.1371/journal.pone.0077783)
Supplement: File S1 — Online Supporting Information. Figure S1. Body mass variation by year for light-bellied Brent geese calculated using 6 years of data. Figure S2. Temporal variation in June NAO from a 60 year dataset. Code S1. R code for Bayesian Hierarchical models used for prediction. Code S2 R code for multivariate response model used to validate the derived variable analyses. (DOCX) [file pone.0077783.s001.docx]

# ONLINE SUPPORTING INFORMATION

**Figure S1**. Caterpillar plot of the year-specific random intercepts calculated from a linear mixed effects model with body mass as the response with a random-intercept term for year. Y axis is shorthand notation for year i.e. 2005/06 is ‘506’. X axis is deviation (in g of mass) from the global mean for all years. There was no evidence that a year-specific intercept was supported (all confidence intervals cross zero).

**Figure S2**. Temporal Trend in June NAO used to detrend the data for subsequent use as predictor of environmental conditions during breeding. June NAO showed a signficiant non-linear trend over time (2nd order polynomial for Year, F_2,60_= 5.82, p=0.005).

**Code S1: Model Code for Bayesian Model Used to Derive 95% Credible Intervals for prediction from top model shown in Figure 1**

The model was run for 100,000 iterations with a thinning interval of 50, after a burnin of 10,000 iterations. The Gelman-Rubin statistic was below 1.05 for all parameters indicating adequate convergence of chains (multivariate psrf = 1.03). Autocorrelation between successive stored iterations was <0.1 for all parameters.

library(runjags)

######################

# Bayesian Analogue of Top Model for Prediction - Number of Offspring

######################

femmod.stage<-"model{

#Intercepts and Priors

for (k in 1:6){

a[k]~dnorm(mu.year,tau.year)

}

mu.year~dnorm(0,0.001)

sd.year~dunif(0,5)

tau.year<-pow(sd.year,-2)

#Slopes

b.naoj~dnorm(0,0.001)

b.seasonmass~dnorm(0,0.001)

b.seasonmass2~dnorm(0,0.001)

bcinaoj~dnorm(0,0.001)

#Model

for (n in 1:213){

juv[n]~dpois(lamb[n])

log(lamb[n])<-a[cycle[n]] + b.seasonmass*seasonmass[n] + b.seasonmass2*seasonmass[n]*seasonmass[n] + b.naoj*naoj[n] + bcinaoj*seasonmass[n]*seasonmass[n]*naoj[n]

#fixef.resid[n]<-juv[n]-lamb[n]

}

# #Predictions

for (k in 1:10){

jpredpoor[k]<-exp(a[2] + b.seasonmass*fakemass[k] + b.seasonmass2*fakemass[k]*fakemass[k] + b.naoj*fakenaojpoor[k] + bcinaoj*fakemass[k]*fakemass[k]*fakenaojpoor[k])

jpredgood[k]<-exp(a[4] + b.seasonmass*fakemass[k] + b.seasonmass2*fakemass[k]*fakemass[k] + b.naoj*fakenaojgood[k] + bcinaoj*fakemass[k]*fakemass[k]*fakenaojgood[k])

# jpredpoor[k]<-exp(mu.year + b.seasonmass*fakemass[k] + b.seasonmass2*fakemass[k]*fakemass[k] + b.naoj*fakenaojpoor[k] + bcinaoj*fakemass[k]*fakemass[k]*fakenaojpoor[k])

# jpredgood[k]<-exp(mu.year + b.seasonmass*fakemass[k] + b.seasonmass2*fakemass[k]*fakemass[k] + b.naoj*fakenaojgood[k] + bcinaoj*fakemass[k]*fakemass[k]*fakenaojgood[k])

}

}"

###Prediction Data

naojmean.neg<-mean(unique(allstage$njs[allstage $njs<0]))

naojmean.pos<-mean(unique(allstage $njs[allstage $njs>0]))

# naojmean.neg<--1

# naojmean.pos<-1

fakenaojgood<-rep(naojmean.neg,10)

fakenaojpoor<-rep(naojmean.pos,10)

fakemass<-seq(-1.5,1.5,length.out=10)

#Data

ringcycs<-data.frame(cyc=(unique(allstage $Year)),cycnum=seq(length(unique(allstage $Year))))

cycle.stage<-ringcycs[,2][match(allstage $Year,ringcycs[,1])]

#Inits and Data for Model

femstagedata<-dump.format(list(seasonmass=allstage$sms,juv= allstage$JuvFuture,cycle=cycle.stage,naoj= allstage $njs,fakemass=fakemass,fakenaojpoor=fakenaojpoor,fakenaojgood=fakenaojgood))

femstageinits<-function(){dump.format(list(b.seasonmass=rnorm(1),b.seasonmass2=rnorm(1),b.naoj=rnorm(1),bcinaoj=rnorm(1),mu.year=rnorm(1),sd.year=runif(1,min=0.01,max=4.99)))}

femstageparams<-c("mu.year","b.seasonmass","b.seasonmass2","b.naoj","bcinaoj","jpredpoor","jpredgood","sd.year","fixef.resid")

femstageburn=10000

femstagesamp=100000

femstagethin=50

femstagejags1<-run.jags(model=femmod.stage,monitor=femstageparams,inits=c(femstageinits(),femstageinits()),burnin=femstageburn,sample=femstagesamp,thin=femstagethin,data=femstagedata)

**Code S2: Model Code for Bivariate Response Model**

**#Simple Prior where nu = matrix dimensions + 1**

priorj2<-list(R=list(V=diag(2),nu=3))

**#Model with Bivariate response**

**#Mass (trait 1) modelled as a function of day of annual cycle (cycleday) and skull size (Skull)**

**#Juveniles (trait 2) Modelled as a Function of June NAO (njs)**

**#As there is only one observation per bird, we fit only an R matrix as an additional random effect of Bird would be unidentifiable (J. Hadfield, pers. comm.)**

mc2<-MCMCglmm(cbind(Mass,JuvFuture)~trait-1 +at.level(trait,1):poly(cycleday,2)+at.level(trait,1):Skull + at.level(trait,2):njs,rcov=~us(trait):units,prior=priorj2,data=allstage,family=c("gaussian","poisson"),verbose=F,nitt=250000,burnin=50000,thin=50)

summary(mc2)

#Return Mean and 95% credible intervals for posterior correlation

posterior.mode(posterior.cor(mc2$VCV)[,2])

HPDinterval(posterior.cor(mc2$VCV)[,2])
